# Probiotics After Metabolic and Bariatric Surgery: A Systematic Review and Meta-Analysis of Randomized Controlled Trials

**DOI:** 10.3390/metabo16060371

**Published:** 2026-05-29

**Authors:** Mohammed Y. Ezzi

**Affiliations:** Department of Surgery, College of Medicine, Jazan University, Jazan 45142, Saudi Arabia; mezzi@jazanu.edu.sa

**Keywords:** probiotics, bariatric surgery, micronutrients, gut dysbiosis

## Abstract

**Background/Objectives:** Patients undergoing metabolic and bariatric surgery (MBS) are at risk of micronutrient deficiencies and gut dysbiosis. Probiotics (such as Lactobacillus, Bifidobacterium) have been proposed as adjunct therapy to optimize postoperative outcomes. This review aimed to evaluate the effect of postoperative probiotic supplementation on anthropometric, metabolic, inflammatory, and micronutrient outcomes in MBS patients. **Methods**: Nine electronic databases were systematically searched, including PubMed, Web of Science, Cochrane Library, Google Scholar, Popline, Global Health Library, Virtual Health Library, New York Academy of Medicine, and OpenGrey, from inception through October 2024. Only randomized controlled trials (RCTs) were included. The Cochrane Collaboration risk-off-bias tool was used for quality assessment. Meta-analyses were performed using Comprehensive Meta-Analysis software version 2. Fixed-effects or random-effects models based on heterogeneity (I^2^ threshold: 50%) were applied. Mean differences (MD) and 95% confidence intervals (CI) were calculated for all continuous variables. **Results**: Thirteen RCTs encompassing 666 patients (probiotics group: n = 344; control group: n = 322) were included. Incomplete outcome data represented the most prevalent high-risk domain (23%). Probiotic supplementation was associated with significantly improved serum vitamin D (MD: 25.32 nmol/L, 95% CI: 6.96–43.67, *p* = 0.007) and vitamin B12 levels (MD: 39.36 pg/mL, 95% CI: 1.88–76.84, *p* = 0.04). No statistically significant differences were observed in anthropometric outcomes (%EWL, BMI, weight, or waist circumference), lipid profile, glycemic indices, or inflammatory markers (TNF-α, IL-6, CRP). **Conclusions**: Postoperative probiotic supplementation may significantly improve vitamin D and B12 levels in patients undergoing MBS, suggesting a supportive role in mitigating micronutrient deficiencies. However, these findings should be interpreted with caution due to substantial heterogeneity across studies. Probiotics did not significantly affect weight loss, metabolic parameters, or inflammatory markers. Clinicians may consider probiotics as an adjunct strategy to support micronutrient status in at-risk postoperative patients. Large-scale, strain-specific trials incorporating standardized dietary control and microbiome profiling are warranted.

## 1. Introduction

Metabolic and bariatric surgery (MBS) represents the most effective and durable treatment for severe obesity, achieving substantial and sustained weight reduction alongside meaningful resolution of obesity-related comorbidities [1]. Despite these well-established benefits, a subset of patients experience suboptimal outcomes, including insufficient weight loss, weight regain, or persistent metabolic disturbances [2]. These challenges have prompted investigation into adjuvant strategies aimed at optimizing postoperative outcomes, with probiotics emerging as a promising candidate given their established ability to modulate gut microbiota composition and function [3].

Obesity is closely associated with gut dysbiosis, a condition characterized by imbalanced microbial diversity that affects up to 40% of individuals with obesity and contributes to metabolic dysfunction through impaired nutrient absorption, altered bile acid metabolism, and dysregulated immune signaling [4,5]. The impact of MBS on the gut microbiome remains controversial: while some evidence suggests that MBS partially restores microbial equilibrium, other studies indicate it may exacerbate existing dysbiosis, particularly in the early postoperative period [6,7].

Given the fundamental role of the gut microbiome in metabolic regulation, immune modulation, and vitamin biosynthesis, probiotics have been proposed as a therapeutic adjunct to enhance post-surgical recovery, reduce infectious complications, and correct micronutrient deficiencies [7,8]. Preclinical and clinical evidence suggests that probiotics may improve anthropometric outcomes, attenuate inflammatory cascades, and restore metabolic balance by re-establishing gut microbial homeostasis in individuals with severe obesity [9].

Multiple randomized controlled trials (RCTs) have examined probiotics as adjunct therapy following various bariatric procedures [10,11,12]. However, the existing literature is characterized by significant heterogeneity in probiotic strains, dosages, follow-up durations, and surgical techniques, limiting the generalizability of individual study findings. Furthermore, the specific role of probiotics in correcting micronutrient deficiencies—a major long-term complication of MBS, particularly deficiencies in vitamin D and B12—has not been comprehensively synthesized.

This analysis was therefore carried out to provide a comprehensive, evidence-based synthesis of the effects of postoperative probiotic supplementation on key clinical endpoints, including anthropometric measurements, lipid and glycemic profiles, inflammatory biomarkers, and micronutrient levels (specifically vitamin D and B12), in patients undergoing MBS.

## 2. Materials and Methods

This systematic review and meta-analysis was performed and reported in accordance with the guidelines of Preferred Reporting Items for Systematic Reviews and Meta-Analyses (PRISMA) [13]. This study exclusively analyzed previously published data; therefore, ethical approval was not necessary. The study protocol was registered with PROSPERO (registration number: CRD42024613302).

### 2.1. Eligibility Criteria

Studies were included in analysis if they: (i) were RCTs (randomized controlled trials); (ii) evaluated the effects of probiotic supplementation in patients undergoing any form of MBS; and (iii) reported at least one of the pre-specified outcomes. No restrictions were applied regarding patient age, sex, ethnicity, comorbidity profile, probiotic strain or formulation, duration of supplementation, or bariatric procedure type. Non-RCT designs, editorials, conference abstracts, book chapters, and studies with incomplete outcome data not retrievable from corresponding authors were excluded.

### 2.2. Search Strategy and Study Selection

A comprehensive literature search was conducted across nine electronic databases: PubMed, Web of Science (ISI), Cochrane, Google Scholar, Popline, Global Health Library, New York Academy of Medicine Grey Literature Report, Virtual Health Library and OpenGrey (SIGLE). Initial searches were performed through August 2022 and updated in October 2024. The search strategy was developed and peer-reviewed using the PRESS (Peer Review of Electronic Search Strategies) checklist.

The PubMed search string was: (“Probiotics”[Mesh] OR “Microbiota”[Mesh] OR probiotics OR probiotic OR “Bacillus coagulans” OR microbiota OR acidophilus OR lactobacillus OR microbiome OR microflora) AND (“Bariatric Surgery”[Mesh] OR “Gastric Bypass”[Mesh] OR “bypass surgery” OR “gastric bypass” OR “bariatric surgery” OR “sleeve gastrectomy” OR “Roux-en-Y Gastric Bypass”). Search strings were adapted for each database using controlled vocabulary and MeSH terms. Duplicates were identified and removed using EndNote X7.4 (Clarivate, London, UK). Full-text review was performed after title and abstract screening using inclusion criteria. Single reviewers completed the selection process on two occasions, one week apart. Any discrepancies were resolved by a senior expert.

### 2.3. Data Extraction

An independent researcher performed the data extraction process using a standardized extraction form, which was piloted on three articles to ensure clarity and consistency. A two-step extraction process was implemented, with a one-week interval between rounds to enhance accuracy and minimize potential errors. Extracted data included study design, patient demographics, surgical procedure, probiotic formulation and dosage, follow-up duration, and outcome data (BMI, waist circumference, percentage excess weight loss, lipid profile, HbA1c, HOMA-IR, vitamin D and B12 levels, and inflammatory markers. The exclusion criteria included non-randomized study design (observational studies, case series, or non-controlled trials), patient population not undergoing metabolic or bariatric surgery, and failure to report at least one pre-specified outcome. For outcomes presented graphically, the PlotDigitizer software version 2.6.8 (SourceForge) was used for data extraction. Data was independently extracted by a reviewer and subsequently verified through cross-checking.

### 2.4. Risk of Bias Assessment 

The Cochrane Collaboration risk-of-bias (RoB) tool for RCTs was used, evaluating seven domains: (1) randomized sequence generation, (2) concealment allocation, (3) blinding the participants, (4) assessment of outcomes conducted in a blinded manner, (5) missing outcome data, (6) selective reporting, and (7) sources of bias. Each domain was rated as low, high, or unclear risk. Studies were classified as high overall risk if two or more domains were rated high risk, or one high-risk domain was accompanied by two or more unclear-risk domains. However, publication bias was not estimated due to the smaller number of studies. According to the Cochrane Handbook, formal assessments of publication bias (such as funnel plots and Egger’s regression test) should only be performed when there are at least 10 studies included in a meta-analysis [14].

### 2.5. Statistical Analysis

Meta-analyses were conducted using Comprehensive Meta-Analysis software version 2 (Biostat Inc., Englewood, NJ, USA). Continuous outcomes were summarized as mean differences or standardized mean differences (SMD) with 95% CI. I^2^ statistic and Cochran Q test were applied for quantifying statistical heterogeneity. A random-effects model (DerSimonian–Laird) was applied when I^2^ > 50%; a fixed-effects model was used when I^2^ ≤ 50%. Publication bias assessment was not performed, as all outcomes included fewer than ten studies, rendering funnel plot analysis and Egger’s regression test statistically unreliable per established methodological guidelines. Statistical significance involved *p* < 0.05.

## 3. Results

**Figure 1:**
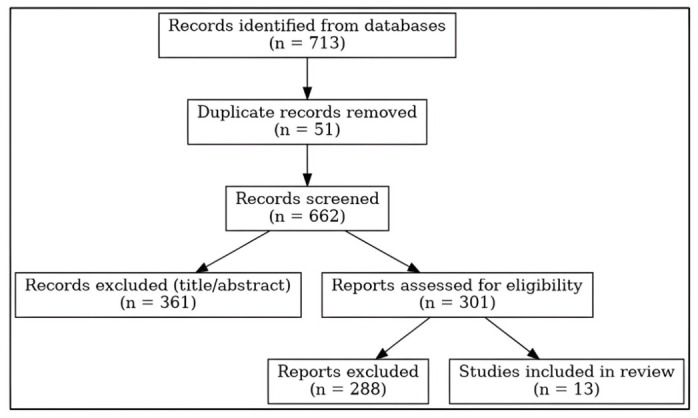
PRISMA flow diagram.

The initial database search retrieved 713 records. Following de-duplication (51 records removed using EndNote), 662 records followed title and abstract screening. Of these, 361 were excluded based on pre-specified criteria. Full-text assessment of 301 records yielded 13 RCTs meeting all eligibility criteria (Figure 1). The 13 included studies enrolled a total of 666 patients: 344 in the probiotic arm and 322 in the control arm. Baseline characteristics and study-level data are presented in Table 1.

### 3.1. Study-Level Risk of Bias Evaluation Results

**Figure 2:**
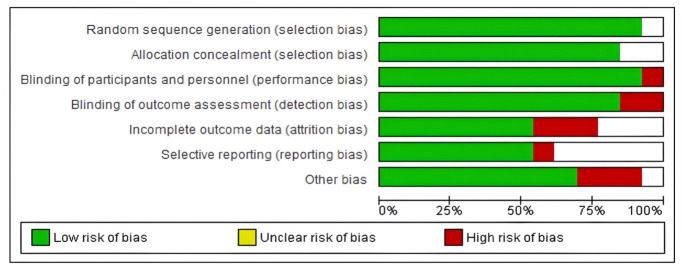
Risk of bias assessment.

Adequate random sequence generation was reported in 12 trials (92%), and allocation concealment was clearly described in 11 trials (85%); both domains were therefore the most frequently rated as low risk. Incomplete outcome data represented the most prevalent high-risk domain, identified in three studies (23%). Risk-of-bias summary graphs are presented in Figure 2.

### 3.2. Anthropometric Outcomes

#### 3.2.1. Percentage Excess Weight Loss (%EWL)

**Figure 3:**
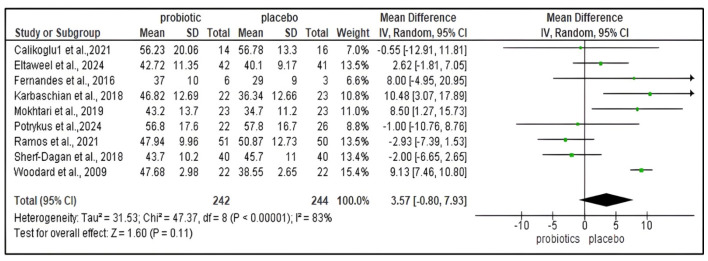
Forest plot of %EWL [15,16,17,18,19,21,22,23,25].

Nine studies (n = 486; probiotics: 242, control: 244) reported %EWL at three months postoperatively. Although the probiotics group demonstrated a numerically higher %EWL, no statistically significant difference between-group was detected (MD = 3.57, 95% CI: −0.80 to 7.93, *p* = 0.11). Substantial heterogeneity was observed (I^2^ = 83%, *p* < 0.00001); a random-effects model was therefore applied (Figure 3).

#### 3.2.2. Body Mass Index (BMI, kg/m^2^)

Nine studies reported postoperative BMI changes (probiotics: n = 260; control: n = 263). No statistically significant difference was detected between groups (MD = −0.52, 95% CI: −1.23 to 0.20, Z = 1.42, *p* = 0.16). Moderate heterogeneity was present (I^2^ = 57%, *p* = 0.02); a random-effects model was used.

#### 3.2.3. Absolute Weight Change (kg)

Eight studies (n = 442; probiotics: 220, control: 222) reported absolute weight change from baseline at three months. No significant between-group difference was found (MD = −1.14 kg, 95% CI: −2.84 to 0.55, Z = 1.32, *p* = 0.19). Substantial heterogeneity was observed (I^2^ = 90%, *p* < 0.00001); a random-effects model was applied.

#### 3.2.4. Waist Circumference (cm)

Seven studies reported waist circumference. No significant difference was identified between probiotics and control groups (MD = −1.48 cm, 95% CI: −4.07 to 1.12, *p* = 0.26). Substantial heterogeneity was present (I^2^ = 88%, *p* < 0.00001); a random-effects model was used.

### 3.3. Lipid Profile

**Figure 4:**
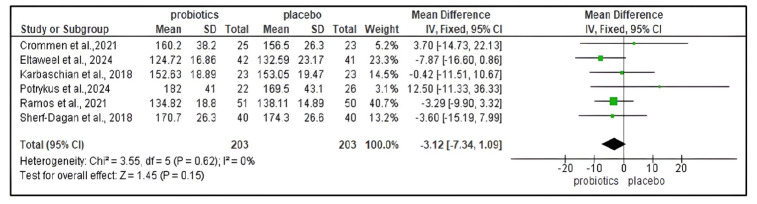
Forest plot of serum cholesterol (mg/dL) across studies [15,17,18,21,22,28].

No significant between-group differences were observed for any lipid parameter. LDL cholesterol (five studies; MD = −1.69 mg/dL, 95% CI: −7.12 to 3.74, *p* = 0.54; I^2^ = 0%), HDL cholesterol (MD = 2.06 mg/dL, 95% CI: −0.46 to 4.57, *p* = 0.11; I^2^ = 16%), triglycerides (five studies; MD = −5.92 mg/dL, 95% CI: −17.04 to 5.20, *p* = 0.30; I^2^ = 0%), and total cholesterol (six studies, n = 406; MD = −3.12 mg/dL, 95% CI: −7.34 to 1.09, *p* = 0.15; I^2^ = 0%) were all comparable between groups. Fixed-effects models were used for all lipid outcomes given low heterogeneity (Figure 4).

### 3.4. Glycemic Profile

Probiotic supplementation did not significantly affect glycemic control. HbA1c (five studies; MD = −0.05%, 95% CI: −0.18 to 0.08, *p* = 0.47; I^2^ = 22%) and HOMA-IR (four studies; MD = 0.26, 95% CI: −0.18 to 0.71, *p* = 0.25; I^2^ = 0%) were both comparable between groups. Fixed-effects models were applied.

### 3.5. Inflammatory Biomarkers

No significant differences between groups were observed for any inflammatory marker. TNF-α (three studies; MD = −3.99 pg/mL, 95% CI: −9.17 to 1.19, *p* = 0.13; I^2^ = 52%), IL-6 (four studies; MD = −0.13 pg/mL, 95% CI: −0.92 to 0.67, *p* = 0.76), and CRP (four studies; MD = 0.87 mg/L, 95% CI: −1.04 to 2.78, *p* = 0.37; I^2^ = 57%) did not significantly differ between the probiotic and control groups.

### 3.6. Micronutrient Levels

#### 3.6.1. Vitamin D (nmol/L)

**Figure 5:**
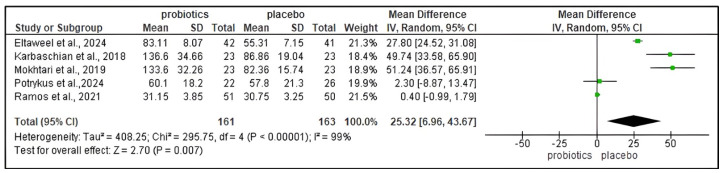
Forest plot of vitamin D (nmol/L) [15,17,19,21,22].

Five studies reported serum vitamin D levels. A random-effects model was applied due to very high heterogeneity (I^2^ = 99%, *p* < 0.00001). Probiotic supplementation was linked with a significant increase in vitamin D (MD = 25.32 nmol/L, 95% CI: 6.96–43.67, Z = 2.70, *p* = 0.007) (Figure 5).

#### 3.6.2. Vitamin B12 (pg/mL)

**Figure 6:**
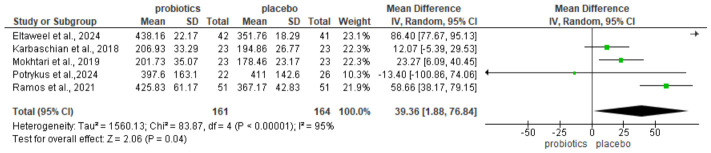
Forest plot of vitamin B12 (pg/mL) [15,17,19,21,22].

Five studies reported serum vitamin B12 levels. Similarly, a random-effects model was applied (I^2^ = 95%, *p* < 0.00001). Probiotic supplementation was associated with a statistically significant increase in vitamin B12 (MD = 39.36 pg/mL, 95% CI: 1.88–76.84, Z = 2.06, *p* = 0.04) (Figure 6).

## 4. Discussion

This systematic review and meta-analysis of 13 RCTs evaluated the adjuvant effects of probiotics on anthropometric, metabolic, inflammatory, and micronutrient outcomes following MBS. The principal finding was that probiotic supplementation was associated with significant improvements in serum vitamin D and vitamin B12 levels, while no significant effects were observed on weight-related, lipid, glycemic, or inflammatory outcomes.

The absence of a significant probiotic effect on anthropometric parameters is consistent with prior evidence. Ramos et al. found no significant between-group difference in %EWL (*p* = 0.290) [15], and Calikoglu et al. similarly reported no significant effect on BMI (*p* = 0.697) [16]. Potrykus et al. demonstrated that both arms showed significant within-group improvements in weight and BMI at one, three, and six months, but without meaningful between-group differences [17]. Sherf-Dagan et al. likewise reported significant reductions in waist circumference in both groups at six months post-sleeve gastrectomy, without a significant intergroup difference (probiotics: −24.1 ± 6.4 vs. placebo: −24.5 ± 6.7 cm; *p* = 0.821) [18]. Suzumura et al. and Swierz et al. reached similar conclusions in adult bariatric populations [29,30]. Mohammadi et al., Daghmouri et al. and Wang et al. conducted meta-analyses and confirmed no significant probiotic effects on anthropometric measures, including BMI, %EWL, and waist circumference [31,32,33]. Collectively, the available evidence suggests that while MBS produces profound weight loss through anatomical and physiological mechanisms, the incremental contribution of probiotics to anthropometric outcomes is minimal, likely because the dominant effects of surgery overshadow subtle microbiome-mediated benefits [34].

Conversely, some studies have reported more favorable outcomes. Borgeraas et al. found greater weight reduction with probiotics compared to placebo in individuals with obesity [35] and Mokhtari et al. reported significant weight loss improvement with probiotics at four months after gastric bypass (*p* = 0.01) [19]. Kim et al. demonstrated that supplementation with *Lactobacillus gasseri* BNR17 reduced the visceral fat and waist circumference in a controlled trial [36]. These discrepancies likely reflect differences in probiotic strain, dose, surgical technique, baseline microbiome composition, and dietary context, all of which may independently modulate microbiota-driven metabolic responses [20,28].

With respect to lipid and glycemic outcomes, no significant probiotic effects were detected in the present analysis. This aligns with findings from Wang et al., who found no significant differences in HbA1c, HOMA-IR, or lipid parameters between probiotic and control groups after bariatric surgery [33]. By contrast, Daghmouri et al. reported a significant probiotic-associated reduction in insulin concentrations without effects on HbA1c [32], and Yao et al. demonstrated significant improvements in HbA1c and fasting insulin with probiotic supplementation in type 2 diabetes patients across 12 RCTs [37]. The discordance across studies is likely attributable to heterogeneity in the gut microbiota, surgical procedure, probiotic strain, and dose, compounded by the limited number of studies per outcome in the current meta-analysis.

The statistically meaningful finding of this meta-analysis is the significant improvement in serum vitamin D and vitamin B12 associated with postoperative probiotic use. Probiotic strains, particularly *Lactobacillus* and *Bifidobacterium* species, may improve intestinal barrier integrity and enhance the bioavailability of fat-soluble and water-soluble vitamins through direct biosynthesis and enhanced absorption [38]. Ramos et al. reported a significant rise in postoperative vitamin D and vitamin B12 concentrations in probiotic-supplemented patients compared to placebo [15], consistent with findings from Woodard et al., who demonstrated greater vitamin B12 levels following Lactobacillus supplementation for 6 months following Roux-en-Y gastric bypass [23].

Probiotics help maintain gut barrier integrity by enhancing the expression and assembly of tight junction proteins (occludin, claudins, ZO-1), thereby reducing intestinal permeability and supporting efficient nutrient absorption and metabolism [39]. Certain Lactobacillus species produce vitamin B12. These species contain genes for de novo B12 synthesis and produce biologically active cobalamin. Some L. plantarum strains also secrete extracellular B12, supporting probiotic use in fermented foods for in situ vitamin B12 fortification [40]. Probiotics may influence bile acid metabolism by modifying the gut microbiome. Intestinal microbiota produces SCFA which regulate secondary bile acids, including lithocholic acid (LCA). LCA acts as an endogenous ligand for the vitamin D receptor (VDR). Its production depends on gut microbial activity and may enhance VDR-mediated signaling. The bile acid–VDR axis may support intestinal homeostasis and may improve vitamin D metabolic efficiency through better receptor activation within the gut–liver system [41]. Furthermore, it has also been observed that perioperative administration of probiotics showed better results in patients undergoing gastrointestinal surgery [42].

The rise in vitamin D observed in this meta-analysis may also partially reflect accelerated release of vitamin D stored in adipose tissue during the rapid weight loss period following surgery [43]. It is important to note that the vitamin D and B12 analyses were characterized by very high heterogeneity (I^2^ = 99% and 95%, respectively), which limits the precision and interpretability of the pooled estimates. This extreme heterogeneity likely reflects differences in baseline micronutrient status, probiotic formulations, surgical procedures, and follow-up duration. Subgroup analyses stratified by surgery type, probiotic strain, or duration of supplementation were not feasible given the limited number of studies included. In addition, the relatively short follow-up duration in most included trials limits the ability to determine whether these improvements are sustained over time or translate into meaningful clinical outcomes. These results should therefore be interpreted with caution, and effect size estimates are best regarded as preliminary.

With respect to inflammatory outcomes, this meta-analysis found no significant probiotic effects on TNF-α, IL-6, or CRP, consistent with findings by Karbaschian et al. [22]. The failure to observe anti-inflammatory effects may reflect insufficient study power, variability in inflammatory measurement methods, or dilution of microbiome-driven effects by the profound systemic anti-inflammatory response induced by weight loss itself.

## 5. Strengths and Limitations

This study adhered to PRISMA 2020 guidelines, was prospectively registered in PROSPERO (CRD42024613302), and employed a comprehensive nine-database search peer-reviewed using the PRESS checklist, with duplicate independent screening and data extraction, reflecting a rigorous methodological approach. To our knowledge, this is the first meta-analysis to specifically evaluate the effects of probiotics on vitamin D and B12 levels following MBS, identifying a clinically meaningful benefit not previously quantified. However, several limitations must be acknowledged. A potential limitation is the inability to formally assess publication bias due to the limited number of included studies (<10). Moreover, a qualitative evaluation suggests a possible risk of bias, as the majority of studies reported favorable outcomes. The presence of unpublished negative or null findings cannot be excluded, which may lead to overestimation of the observed effects. The follow-up period was brief and heterogeneous, precluding conclusions about long-term effects. The considerable variability in probiotic strains, doses, and formulations prevented subgroup or sensitivity analyses and may mask strain-specific effects. Inadequate reporting of placebo composition and the absence of dietary control across most included trials introduce potential confounding, while the lack of microbiome profiling limits mechanistic interpretation of the observed micronutrient benefits. Baseline vitamin D and B12 status, as well as concomitant supplement use, are potential confounders in bariatric patients that could influence observed changes. Additionally, increases in serum vitamin D may partly result from release of stored vitamin D due to postoperative fat loss, rather than probiotic supplementation alone. Formal certainty-of-evidence assessment using the GRADE framework was not performed in this review, given the limited number of studies per outcome and the exploratory nature of the synthesis. Consequently, the overall confidence in the pooled estimates and the robustness of the conclusions may be reduced, particularly in the context of clinical decision-making. Due to the involvement of a single reviewer, there may be a risk of selection and extraction bias. Although a two-step process was employed to improve reliability, the absence of independent verification may affect the robustness and reproducibility of the extracted data. Additionally, subjective judgment during data interpretation cannot be entirely excluded, potentially influencing the consistency of extracted variables. Future research should focus on strain-specific probiotic trials to identify the most effective bacterial strains for targeted clinical outcomes. Additionally, microbiome-guided therapeutic approaches and well-designed, standardized randomized controlled trials with strict dietary control are needed to improve consistency and clinical applicability of findings.

## 6. Conclusions

This review provides evidence that postoperative probiotic supplementation significantly improves serum vitamin D and vitamin B12 levels in patients undergoing MBS, without producing significant effects on weight-related, metabolic, or inflammatory outcomes. These findings support a targeted clinical role for probiotics in addressing post-bariatric micronutrient deficiencies, particularly in patients at heightened risk for vitamin D and B12 insufficiency. The integration of probiotics into postoperative bariatric care protocols warrants consideration, with the caveat that further evidence from large-scale, strain-specific, diet-controlled RCTs incorporating microbiome profiling is required to define optimal supplementation strategies and identify which patient subgroups may get maximum benefit.

## Figures and Tables

**Table 1:** Baseline and demographic characteristics of included studies.

Author, Year	Country	Age (Years)	BMI (kg/m^2^)	N (Probiotics/Placebo)	Surgery Type	Probiotic Strain(s)	Follow-Up
Ramos et al., 2021 [15]	Brazil	40.5	44.1	50/51	RYGB	*Lactobacillus acidophilus* NCFM; *Bifidobacterium lactis* Bi-07	12 weeks
Calikoglu et al., 2021 [16]	Turkey	18–65 (range)	50.6	14/16	RYGB	Yogurt (probiotic)	6 months
Potrykus et al., 2024 [17]	Poland	41.6	43.4	22/26	RYGB or LSG	SANPROBI^®^ Barrier (9 strains)	12 weeks
Sherf-Dagan et al., 2018 [18]	Israel	41.9	42.3	40/40	LSG	Combination of 11 strains (*Lactobacillus*, *Bifidobacterium*, Lactococcus lactis, *Streptococcus thermophilus*)	12 months
Mokhtari et al., 2019 [19]	Iran	34.7	44.7	23/23	Mini Gastric Bypass	Combination of 7 strains (*Lactobacillus*, *Streptococcus* *thermophilus*, *Bifidobacterium*)	12 months
Crommen et al., 2020 [20]	Germany	40	44	25/23	Mini Gastric Bypass	Multi-strain probiotic powder (tailored)	12 weeks
Mohamadain & Eltaweel, 2024 [21]	Egypt	41.4	44.3	42/41	LSG	*Lactobacillus acidophilus*; *Bifidobacterium animalis*	12 weeks
Karbaschian et al., 2018 [22]	Iran	34.7	44.8	22/23	Mini Gastric Bypass (OAGB-MGB)	Combination of 7 strains (*Lactobacillus*, *Streptococcus* *thermophilus*, *Bifidobacterium*)	16 weeks
Woodard et al., 2009 [23]	USA	44.9	47.7	22/22	RYGB	*Lactobacillus* species	6 months
Komorniak et al., 2023 [24]	Poland	44.7	31.2	21/17	RYGB or LSG	Combination of 9 strains (SANPROBI^®^ Barrier)	5 weeks
Fernandes et al., 2016 [25]	Brazil	36.9	40.0	6/3	RYGB	Combination of 4 strains + *prebiotic* (*Lactobacillus*, *Bifidobacterium*)	12 weeks
Chen et al., 2016 [26]	Taiwan	35.1	29.3	37/16	RYGB	*Clostridium butyricum* (*n* = 19) or *Bifidobacterium longum* (*n* = 18)	2 weeks
Ghafouri-Taleghani et al., 2024 [27]	Iran	39.1	34.0	20/21	RYGB or LSG	Combination of 7 strains	12 weeks

## Data Availability

The original contributions presented in this study are included in the article.

## Abbreviations

The following abbreviations are used in this manuscript:

BMI	Body Mass Index
MBS	Metabolic and Bariatric Surgery
MD	Mean Difference
RCT	Randomized Controlled Trial
RYGB	Roux-en-Y Gastric Bypass
%EWL	Percentage Excess Weight Loss
